# Ultrasound-Assisted Extraction (UAE) and Solvent Extraction of Papaya Seed Oil: Yield, Fatty Acid Composition and Triacylglycerol Profile

**DOI:** 10.3390/molecules181012474

**Published:** 2013-10-10

**Authors:** Shadi Samaram, Hamed Mirhosseini, Chin Ping Tan, Hasanah Mohd Ghazali

**Affiliations:** 1 Department of Food Technology, Faculty of Food Science and Technology, University Putra Malaysia, Serdang 43400, Selangor, Malaysia; E-Mails: shsamaram@gmail.com (S.S.); tancp@putra.upm.edu.my (C.P.T.); 2 Department of Food Science, Faculty of Food Science and Technology, University Putra Malaysia, Serdang 43400, Selangor, Malaysia; E-Mail: hasanah@putra.upm.edu.my

**Keywords:** ultrasound-assisted extraction, solvent extraction, papaya seed oil, fatty acid composition, triacylglycerol profile, LC-MS

## Abstract

The main objective of the current work was to evaluate the suitability of ultrasound-assisted extraction (UAE) for the recovery of oil from papaya seed as compared to conventional extraction techniques (*i.e*., Soxhlet extraction (SXE) and solvent extraction (SE)). In the present study, the recovery yield, fatty acid composition and triacylglycerol profile of papaya seed oil obtained from different extraction methods and conditions were compared. Results indicated that both solvent extraction (SE, 12 h/25 °C) and ultrasound-assisted extraction (UAE) methods recovered relatively high yields (79.1% and 76.1% of total oil content, respectively). Analysis of fatty acid composition revealed that the predominant fatty acids in papaya seed oil were oleic (18:1, 70.5%–74.7%), palmitic (16:0, 14.9%–17.9%), stearic (18:0, 4.50%–5.25%), and linoleic acid (18:2, 3.63%–4.6%). Moreover, the most abundant triacylglycerols of papaya seed oil were triolein (OOO), palmitoyl diolein (POO) and stearoyl oleoyl linolein (SOL). In this study, ultrasound-assisted extraction (UAE) significantly (*p* < 0.05) influenced the triacylglycerol profile of papaya seed oil, but no significant differences were observed in the fatty acid composition of papaya seed oil extracted by different extraction methods (SXE, SE and UAE) and conditions.

## 1. Introduction

In recent years, bio-recovery of valuable byproducts from agro-biomass wastes and underutilized products has become noticeable [1]. Most fruit processing units have massive disposal of biomass waste issues (*i.e*., seeds, skin, pulp, *etc*.). Several studies [2,3] have suggested the bio-recovery of different byproducts like enzymes, essential oils, ethanol and pharmaceuticals from fruit wastes (mango, banana, pineapple and papaya). Papaya fruit is a high yielding crop from the family *Caricaceae* of the genus *Carica* that now grows in many tropical countries. However, *Carica* papayas are originally from tropical and subtropical America and Africa. Malaysian farms produce about 72,000 tons of papaya each year [4]. *Sekaki* papaya is mostly available papaya variety in Malaysian local markets that has medium size fruits with 1.5–2 kg weight. Papaya seeds constitute 15%–20% mass of fruit that represent a considerable amount of papaya fruit waste in processing units [4]. Papaya seeds have the potential to produce 30%–34% oil with nutritional and functional properties highly similar to olive oil [5,6,7]. However, the edibility of papaya seed oil has not been confirmed by previous studies [5]. Different varieties of papaya fruits are different in seeds, size, shape and flavor [7,8].

Different extraction methods (*i.e*., solvent extraction, aqueous enzymatic extraction and extrusion expelling process) have been examined for the recovery of papaya seed oil in previous studies [5,6]. Furthermore, the optimization of extraction conditions (*i.e*., time, temperature, solvent type, solid to solvent ratio and particle size) in solvent extraction methods has been studied in order to obtain extra yield and better quality index of the product under the optimum extraction conditions [9,10]. Ultrasound-assisted extraction (UAE) method has been used as an alternative for solvent extraction due to several advantages it offers (*i.e*., simplicity, inexpensive equipment, and remarkable reduction in solvent amount, temperature and time of extraction) [11]. Previous researchers applied the ultrasound technique for the extraction of oil and bioactive compounds from different plant sources [12,13]. In the current study, the effect of three different extraction methods on the fatty acid composition (FAC) and triacylglycerol (TAG) profile of papaya seed oil were investigated. The idea was to evaluate the suitability of ultrasound-assisted extraction (UAE) as compared to conventional extraction methods (*i.e*., Soxhlet extraction (SXE) and solvent extraction (SE)). It was hypothesized that ultrasound-assisted extraction (UAE) might be a more efficient method for the recovery of papaya seed oil than conventional extraction methods. The efficiencies of different extraction techniques were assessed by determining the recovery yield, fatty acid composition (FAC), and triacylglycerol (TAG) profile of papaya seed oil obtained under different extraction conditions.

## 2. Results and Discussion

### 2.1. Yield and Fatty Acid Composition of Differently Extracted Papaya Seed Oils

**Figure 1:**
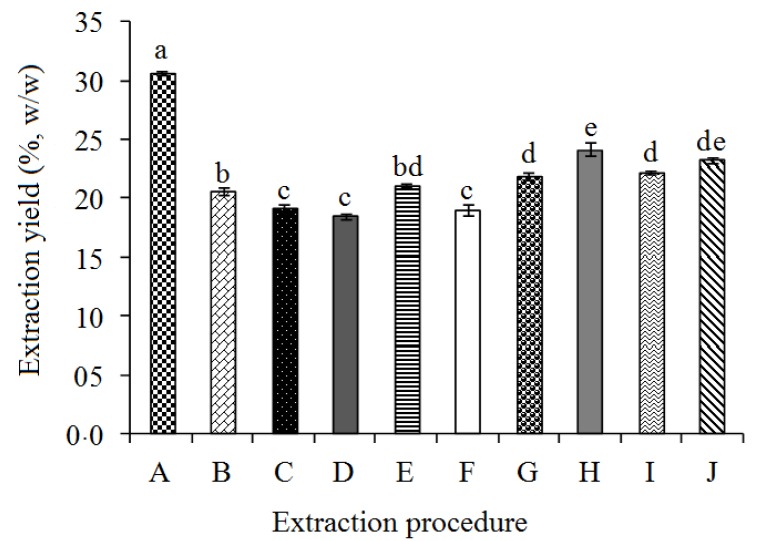
Recovery yield (%, W/W) of papaya seed oil as function of extraction methods and conditions.

The oil recovery yields of different extraction methods and conditions are compared in Figure 1. Results indicated that *Sekaki* papaya seeds had 30.4% oil content based on Soxhlet extraction. Solvent extraction (SE, 12 h/25 °C) and ultrasound-assisted extraction (UAE) recovered a relatively high yield to the 79.1% and 76.1% of total oil content, respectively.

(^a–e^, the significant difference at the confidence level of 95%, (mean ± SD, *n* = 3); A: Soxhlet extraction (SXE); B: solvent extraction, SE (3 h/25 °C); C: SE (3 h/50 °C); D: SE (6 h/25 °C); E: SE (6 h/50 °C); F: SE (9 h/25 °C); G: SE (9 h/50 °C); H: SE (12 h/25 °C); I: SE (12 h/50 °C); J: ultrasound-assisted extraction (UAE).

This might be due to the long extraction time used in the solvent extraction and high ultrasound power in the ultrasound-assisted extraction. Results indicated that, oil recovery percentages were increased by prolonging the extraction time and elevating the extraction temperature. Puangsri *et al*. [5] reported that, aqueous enzymatic extraction at 45 °C for 24 h under continuous shaking conditions resulted in a relatively high extraction yield for papaya seed oil (~78.8% of total oil content). However, a low extraction yield (13.9%–15.2% of total oil content) was reported for screw pressed extracted papaya seed oil in another survey [6]. This confirms the significant effect of extraction method and conditions on the recovery yield of papaya seed oil. The present study showed that, ultrasound-assisted extraction could recover a considerable amount of papaya seed oil in short extraction time (30 min) as compared to SE and SXE and other extraction methods reported by previous researchers [5,6]. The oil content of papaya seed was comparable with the oil recovery from different fruit seeds (Table 1). Olive oil and grape seed oil are commercially produced in different countries [14,15]. The present study revealed that papaya seeds had relatively higher oil content (30%–34%) than olive (22%–24%) [14] as well as grape seed (8%–15%) [15]. On the other hand, it showed lower oil content than watermelon and pumpkin seeds. As papaya seeds contribute the considerable waste amount in fruit processing units in tropical countries like Malaysia [4], it could be utilized as an inexpensive raw material for production of commercial papaya seed oil.

**Table 1:** Oil from different fruit sources.

Oil Source	Oil content %	PUFA ^1^%	MUFA ^2^%	SFA ^3^%	Reference
Papaya seed oil	30–34	2.1–6.3	67.5–77.6	18.6–29.0	Puangsri *et al*. [5]; Lee *et al*. [6]
Olive oil ^4^	22–24	3.5–22.5	55.3–86.5	10.5–20.0	Salvador *et al*. [14]; Codex [16]
Grape seed oil	8–15	50.0–83.0	13.7–36.5	5.8.0–23.5	Passos *et al*. [15]; Shahidi [17]
Orange seed oil	32–35	43.5–45.0	26.0–27.0	28.0–29.0	Shahidi [17]; Ajewole [18]
Apple seed oil	21–24	48.4–65.3	24.7–43.0	6.3–12.4	Shahidi [17]; Tian *et al*. [19]
Watermelon seed oil	50	60.0	18.4	21.6	Baboli and Kordi [20]
Pumpkin seed oil	42–45	55.6	20.8	23.5	Shahidi [21]; Schinas *et al*. [22]

^1^ PUFA, polyunsaturated fatty acids; ^2^ MUFA, monounsaturated fatty acids; ^3^ SFA, saturated fatty acids; ^4^ olive oil from the fresh fruit.

**Figure 2:**
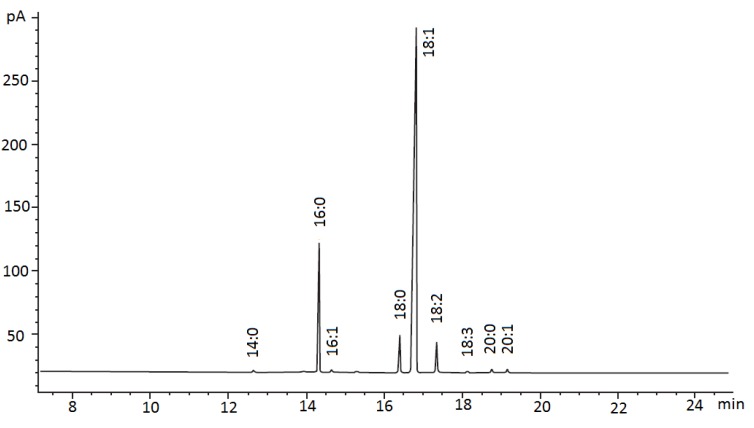
GC-FID chromatogram indicated the fatty acid compositions (FAC) of papaya seed oil from solvent extraction.

Figure 2 shows the fatty acid composition (FAC) of solvent-extracted papaya seed oil. FAC analysis revealed that, the predominant fatty acids in papaya seed oil were oleic (18:1, 70.5%–74.7%), palmitic (16:0, 14.9%–17.9%), stearic (18:0, 4.50%–5.25%) and linoleic acid (18:2, 3.63%–4.60%) (Table 2). Other minor fatty acids (such as myristic acid (14:0), palmitoleic acid (16:1), linolenic acid (18:3), arachidic acid (20:0) and gadoleic acid (20:1)) were less than 0.5% in papaya seed oil (Table 2).

In this study, *Sekaki* variety papaya seed oil showed a slightly different fatty acid composition (FAC) as compared to the fatty acid composition of papaya seed oil reported by previous researchers [5,6]. The variations could be due to the different extraction methods used and the utilization of different papaya varieties in previous studies. As shown in Table 1, the fatty acid composition of papaya seed oil was similar to that of olive oil [14,16]. Papaya seed oil had a lower level of PUFA than grape seed oil (50.0%–83.0%) [15], orange seed oil (43.5%–45.0%) [17,18], apple seed oil (48.4%–65.3%) [19], watermelon seed oil (59.6%) [20] and pumpkin seed oil (55.6%) [21,22]. Both olive oil and papaya seed oil are rich sources of oleic acid (18:1), which is beneficial for the human body. Moreover, oleic acid is the indicator of high stability in frying oils [23,24].

**Table 2:** Fatty acid composition of differently extracted papaya seed oils.

Extraction	Fatty Acids									
	14:0	16:0	16:1	18:0	18:1	18:2	18:3	20:0	20:1	Others
SXE ^1^	0.21 ± 0.02 ^a^	14.9 ± 0.05 ^a^	0.27 ± 0.04 ^a^	5.21 ± 0.01 ^a^	74.2 ± 0.21 ^a^	3.50 ± 0.03 ^a^	0.17 ± 0.01 ^a^	0.38 ± 0.01 ^a^	0.42 ± 0.03 ^a^	0.74 ± 0.02 ^a^
UAE ^2^	0.19 ± 0.01 ^a^	15.1 ± 0.06 ^a^	0.27 ± 0.00 ^a^	5.12 ± 0.00 ^a^	74.2 ± 0.09 ^a^	3.54 ± 0.03 ^a^	0.15 ± 0.00 ^a^	0.37 ± 0.00 ^a^	0.40 ± 0.01 ^a^	0.57 ± 0.02 ^b^
SE ^3^ (3 h/25 °C)	0.19 ± 0.00 ^a^	15.3 ± 0.06 ^a^	0.28 ± 0.00 ^a^	5.13 ± 0.00 ^a^	74.2 ± 0.06 ^a^	3.51 ± 0.02 ^a^	0.15 ± 0.00 ^a^	0.36 ± 0.01 ^a^	0.40 ± 0.01 ^a^	0.48 ± 0.03 ^c^
SE (3 h/50 °C)	0.19 ± 0.01 ^a^	15.2 ± 0.20 ^a^	0.28 ± 0.00 ^a^	5.23 ± 0.02 ^a^	74.4 ± 0.40 ^a^	3.61 ± 0.03 ^a^	0.15 ± 0.00 ^a^	0.41 ± 0.00 ^a^	0.42 ± 0.01 ^a^	0.11 ± 0.01 ^d^
SE (6 h/25 °C)	0.20 ± 0.00 ^a^	15.3 ± 0.10 ^a^	0.30 ± 0.00 ^a^	5.00 ± 0.09 ^a^	74.6 ± 0.23 ^a^	3.60 ± 0.05 ^a^	0.16 ± 0.00 ^a^	0.41 ± 0.01 ^a^	0.41 ± 0.01 ^a^	0.02 ± 0.0 ^e^
SE (6 h/50 °C)	0.18 ± 0.02 ^a^	15.3 ± 0.14 ^a^	0.27 ± 0.01 ^a^	5.14 ± 0.04 ^a^	74.5 ± 0.23 ^a^	3.63 ± 0.03 ^a^	0.15 ± 0.00 ^a^	0.40 ± 0.00 ^a^	0.41 ± 0.00 ^a^	0.02 ± 0.0 ^e^
SE (9 h/25 °C)	0.20 ± 0.01 ^a^	15.1 ± 0.06 ^a^	0.31 ± 0.01 ^a^	5.12 ± 0.05 ^a^	74.4 ± 0.26 ^a^	3.59 ± 0.01 ^a^	0.16 ± 0.01 ^a^	0.39 ± 0.03 ^a^	0.42 ± 0.01 ^a^	0.31 ± 0.01 ^e^
SE (9 h/50 °C)	0.20 ± 0.00 ^a^	15.4 ± 0.01 ^a^	0.30 ± 0.00 ^a^	5.25 ± 0.01 ^a^	74.2 ± 0.06 ^a^	3.63 ± 0.06 ^a^	0.16 ± 0.00 ^a^	0.41 ± 0.01 ^a^	0.41 ± 0.00 ^a^	0.04 ± 0.0 ^e^
SE (12 h/25 °C)	0.19 ± 0.00 ^a^	15.1 ± 0.10 ^a^	0.28 ± 0.00 ^a^	5.14 ± 0.02 ^a^	74.3 ± 0.03 ^a^	3.54 ± 0.01 ^a^	0.15 ± 0.00 ^a^	0.37 ± 0.00 ^a^	0.40 ± 0.00 ^a^	0.53 ± 0.02 ^c^
SE (12 h/50 °C)	0.18 ± 0.01 ^a^	15.2 ± 0.10 ^a^	0.28 ± 0.00 ^a^	5.21 ± 0.01 ^a^	74.5 ± 0.16 ^a^	3.59 ± 0.02 ^a^	0.15 ± 0.00 ^a^	0.40 ± 0.00 ^a^	0.41 ± 0.01 ^a^	0.08 ± 0.02 ^f^

^a,b^ indicated the significant difference at the confidence level of p < 0.05. (Mean ± SD, n = 2); ^1^ SXE (Soxhlet extraction); ^2^ UAE (ultrasound-assisted extraction); ^3^ SE (solvent extraction); 14:0, myristic acid; 16:0, palmitic acid; 16:1, palmitoleic acid; 18:0, stearic acid; 18:1, oleic acid; 18:2, linoleic acid; 18:3, linolenic acid; 20:0, arachidic acid; 20:1, eicosenoic acid.

### 2.2. Triacylglycerol Profile of Differently Extracted Papaya Seed Oils

**Figure 3:**
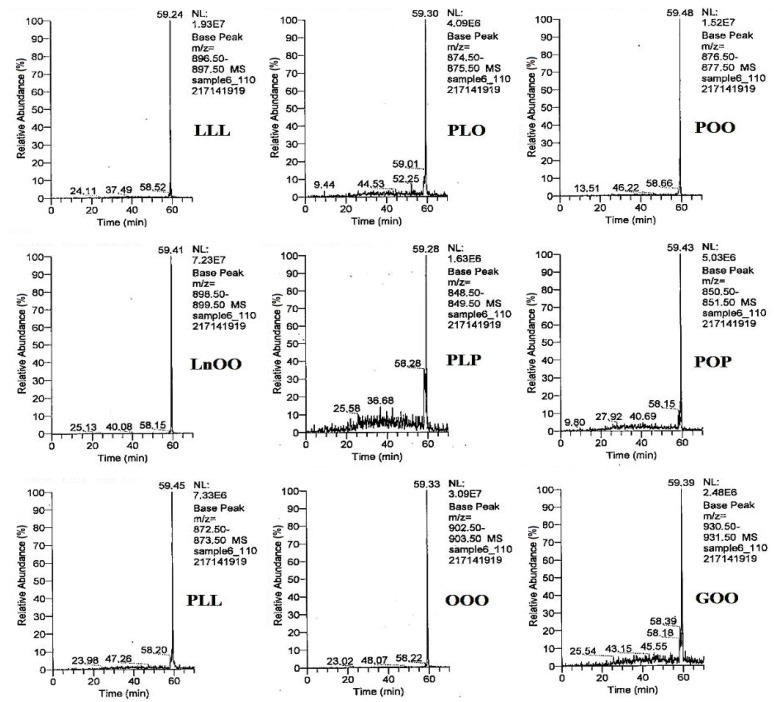
LC-MS chromatograms indicated triacylglycerol (TAG) profile of papaya seed oil in separate chromatograms (LLL: trilinolein; PLO: palmitoyl linoleoyl olein; POO: palmitoyl diolein; LnOO: linolenoyl diolein; PLP: dipalmitoyl linolein; POP: dipalmitoyl olein; PLL: palmitoyl dilinolein; OOO: triolein; GOO: gadoyl-diolein).

Qualitative analysis of the triacylglycerol (TAG) profile of papaya seed oil was carried out by a liquid chromatography-mass spectrometry (LC-MS) method (Figure 3). The quantification analysis was carried out by a high performance liquid chromatography (HPLC). 

**Figure 4:**
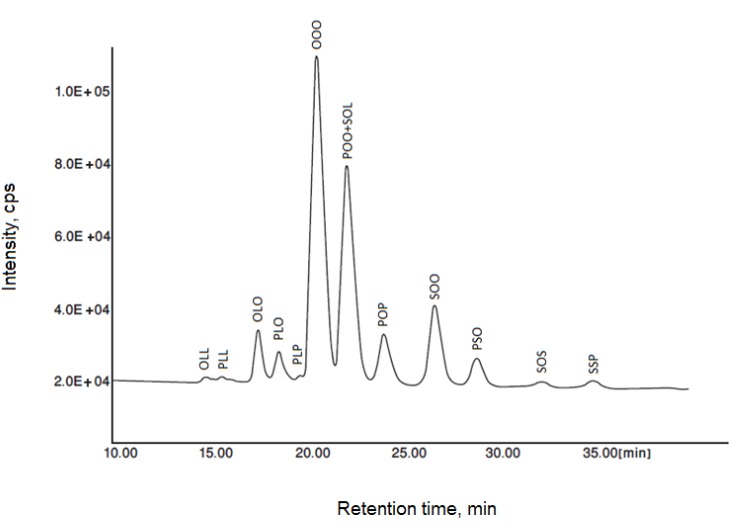
The representative triacylglycerol (TAG) profile of solvent extracted papaya seed oil (OLL: oleoyl dilinolein; PLL: palmitoyl dilinolein; OLO: linoleoyl diolein; PLO: palmitoyl linoleoyl olein; PLP: linoleoyl dipalmitin; OOO: triolein; POO: palmitoyl diolein; SOL: stearoyl oleoyl linolein; POP: dipalmitoyl olein; SOO: stearoyl diolein; PSO: palmitoyl stearoyl olein; SOS: distearoyl olein; SSP: distearoyl palmitin).

The most abundant triacylglycerols in papaya seed oil were triolein (OOO), palmitoyl diolein (POO), stearoyl oleoyl linolein (SOL), stearoyl diolein (SOO), dioleoyl linolein (LOO), dipalmitoyl olein (POP), palmitoyl stearoyl olein (PSO) and palmitoyl linoleoyl olein (PLO) (Figure 3 and Figure 4). In addition, some of the triacylglycerols (such as, OLL, PLL, PLP, SOS, SSP) appeared in minor amounts in the HPLC chromatogram, and other triacylglycerols (*i.e*., LLL, GLO, LnOO, GOO, AOO, BOO) were only detectable by LC-MS. The identified triacylglycerols were obtained in the last 10 min of the LC-MS run, where the pure non-polar solvent was used as a mobile phase.

The triacylglycerol profile of papaya seed oil obtained by different extraction methods (*i.e*., solvent extraction and screw press extraction) from Sekaki seed and two different papaya varieties (Batek Batu and Tainoung) are displayed in Table 3. *Sekaki* seed oil (solvent extraction) had higher levels of OOL and POL than papaya seed oil from *Batek Batu* and *Tainoung* varieties. It also showed lower contents of OOO, POO and SOL than *Batek Batu* and *Tainoung* seed oils (Table 3). This might be due to the utilization of different varieties and extraction techniques. Table 4 also displays the triacylglycerol profile of differently extracted papaya seed oils. The distribution of triacylglycerols affects the physical properties of the oil (such as crystal structure and melting behavior) [25]. In this study, differently extracted papaya seed oils contained 37%–42% OOO (Table 3).

**Table 3:** Triacylglycerol levels in papaya seed oil from different papaya varieties.

Triacylglycerol (TAG)	Papaya fruit varieties		
	*Sekaki* ^1^	*BatekBatu* ^2^	*Tainoung* ^3^
OOL	4.40	3.70	2.54
POL	2.80	2.30	1.72
OOO	41.3	44.6	43.8
POO + SOL	27.7	30.5	33.8
PPO	6.15	5.10	6.19
SOO	9.70	9.80	8.37
POS	3.15	3.80	2.41
Others	4.8	0.2	1.17

^1^ Soxhlet-extracted papaya seed oil in the present study; ^2^ solvent-extracted papaya seed oil; ^3^ screw-pressed extracted papaya seed oil; OOL: dioleoyl linolein; POL: palmitoyl oleoyl linolein; OOO: triolein; POO: dioleoyl palmitin; SOL: stearoyl oleoyl linolein; PPO: dipalmitoyl olein; SOO: dioleoyl stearin; POS: palmitoyl oleoyl stearin.

**Table 4:** Triacylglycerol profile of differently extracted papaya seed oils.

Extraction	Triacylglycerol area%							
	OOL	POL	OOO	POO + SOL	PPO	SOO	POS	Others
SXE ^1^	4.40 ± 0.14 ^a^	2.80 ± 0.14 ^a^	41.3 ± 1.84 ^a^	27.7 ± 1.84 ^a^	6.15 ± 0.21 ^a^	9.70 ± 0.42 ^a^	3.15 ± 0.10 ^a^	4.80 ± 0.23 ^a^
UAE ^2^	4.90 ± 0.30 ^a^	3.80 ± 0.30 ^c^	37.0 ± 0.00 ^b^	25.3 ± 0.42 ^b^	5.90 ± 0.21 ^a^^,b^	8.40 ± 0.14 ^b^	2.75 ± 0.10 ^c^	11.95 ± 0.50 ^b^
SE ^3^ (3 h/25 °C)	4.35 ± 0.10 ^a^	2.75 ± 0.10 ^a^	42.2 ± 0.00 ^a^	29.0 ± 0.00 ^a^	6.40 ± 0.00 ^a^	10.0 ± 0.00 ^a^	3.25 ± 0.10 ^a^	2.05 ± 0.30 ^c^
SE (3 h/50 °C)	4.35 ± 0.10 ^a^	2.70 ± 0.00 ^a^	42.0 ± 0.00 ^a^	28.8 ± 0.35 ^a^	6.25 ± 0.10 ^a^	10.0 ± 0.10 ^a^	3.30 ± 0.00 ^a^	2.60 ± 0.40 ^c^
SE (6 h/25 °C)	4.45 ± 0.10 ^a^	2.90 ± 0.00 ^a^	41.0 ± 1.40 ^a^	27.2 ± 1.20 ^a^^,b^	5.60 ± 0.30 ^b^	10.0 ± 0.42 ^a^	3.35 ± 0.10 ^a^^,b^	5.5 ± 0.7 ^a^
SE (6 h/50 °C)	4.10 ± 0.30 ^a^	2.45 ± 0.20 ^a^^,b^	41.7 ± 0.42 ^a^	28.2 ± 0.14 ^a^	6.20 ± 0.00 ^a^	10.0 ± 0.00 ^a^	3.40 ± 0.00 ^a^	3.95 ± 0.6 ^a^
SE (9 h/25 °C)	4.25 ± 0.10 ^a^	2.80 ± 0.14 ^a^	41.0 ± 1.40 ^a^	27.7 ± 0.90 ^a^	5.75 ± 0.20 ^a^^,b^	9.80 ± 0.42 ^a^	3.20 ± 0.14 ^a^	5.5 ± 0.5 ^a^
SE (9 h/50 °C)	4.00 ± 0.00 ^a^	2.40 ± 0.00 ^a^^,b^	42.3 ± 0.14 ^a^	28.2 ± 0.14 ^a^	6.20 ± 0.00 ^a^	10.0 ± 0.10 ^a^	3.30 ± 0.00 ^a^	3.6 ± 0.5 ^a^
SE (12 h/25 °C)	4.25 ± 0.10 ^a^	2.80 ± 0.00 ^a^	42.4 ± 0.14 ^a^	28.3 ± 0.10 ^a^	6.10 ± 0.00 ^a^	10.0 ± 0.00 ^a^	3.35 ± 0.10 ^a^	2.8 ± 0.4 ^c^
SE (12 h/50 °C)	3.65 ± 0.90 ^a^	2.60 ± 0.00 ^a^	42.15 ± 0.10 ^a^	28.6 ± 0.00 ^a^	6.25 ± 0.10 ^a^	10.0 ± 0.00 ^a^	3.30 ± 0.00 ^a^	3.45 ± 0.5 ^a^

^a^^,^^c^ indicated the significant difference at the confidence level of *p* < 0.05 (Mean ± SD, *n* = 2); ^1^ SXE (Soxhlet extraction); ^2^ UAE (ultrasound-assisted extraction); ^3^ SE (solvent extraction); OOL: dioleoyl linolein; POL: palmitoyl oleoyl linolein; OOO: triolein; POO: dioleoyl palmitin; SOL: stearoyl oleoyl linolein; PPO: dipalmitoyl olein; SOO: dioleoyl stearin; POS: palmitoyl oleoyl stearin.

OOO is the main triacylglycerol in extra virgin olive oil (23%–48%). It contributes to the high stability and health benefits of the oil [23,26]. Results showed that solvent-extracted papaya seed oils had the highest levels of OOO (41.0%–42.4%) among all samples (Table 4). On the other hand, the ultrasound-assisted extraction significantly affected the level of triacylglycerols in papaya seed oil. Ultrasound-extracted papaya seed oil exhibited the lowest significant (*p* < 0.05) level of OOO (37.0 ± 0.00) and POO + SOL (25.3 ± 0.42). This might be due to the fact that, the ultrasound-assisted extraction was performed in shorter extraction times (30 min) than SE and SXE. Results revealed that different solvent extraction conditions (SE, SXE) did not significantly (*p* > 0.05) affect the level of SOO. However, extraction method (UAE) significantly (*p* < 0.05) influenced the level of SOO in triacylglycerol profile of papaya seed oil (Table 4). The current study revealed that the glycerol esters OOL, POL and POS were detected at less than 5% in differently extracted papaya seed oils. The levels of OOL (3.70%–4.90%) were not significantly (*p* > 0.05) affected by the extraction methods and conditions. The lowest significant (*p* < 0.05) amount of POS was detected in ultrasound-extracted papaya seed oil (2.75 ± 0.10). 

## 3. Experimental

### 3.1. Chemicals and Materials

Rippened *Sekaki* papaya fruits were purchased from a hypermarket (Selangor, Malaysia). The pure standards of fatty acid methyl esters (FAME) were obtained from Sigma-Aldrich (St. Louis, MO, USA). In this study, *n*-hexane (reagent grade and HPLC grade), petroleum ether and methanol, 2-propanol, acetone and acetonitrile (HPLC grade) were purchased from Fisher Scientific (Pittsburgh, PA, USA). Ethyl alcohol 96% was supplied by Merck (Darmstadt, Germany).

### 3.2. Sample Preparation

Ripened papaya fruits were chosen according to their maturity stages [7,27]. Fruits were cleaned and cut into halves in order to collect the seeds. Collected seeds were washed and dried in 45 °C oven for 2 days. The dried seeds were ground to the powder form and sieved to achieve uniform particle size. The seed powder was kept in 4 °C refrigerator until extraction.

### 3.3. Solvent Extraction

Oil extraction was carried out according to AOCS Official Method (Am 2-93) [28,29]. Hexane was utilized as the extraction solvent in all extraction procedures. In this study, 10 g seed powder from two different papaya varieties was subjected for Soxhlet extraction (SXE). For solvent extraction (SE), seed powder was mixed with solvent (1:10 g/mL) in a blue cap bottle and the bottles were covered with aluminum foil. In this study, solvent extraction process (SE) was performed under different experimental conditions (*i.e*., time: 3, 6, 9, and 12 h; temperature: 25 and 50 °C). All bottles were shaken by a temperature-controllable water bath shaker at 100 rpm under different extraction conditions [10,30]. All extractions were performed in triplicate.

### 3.4. Ultrasound-Assisted Extraction (UAE)

In the current work, ultrasound-assisted extraction (UAE) was also applied for the recovery of oil from *Sekaki* variety seeds. An ultrasonic water bath (Power sonic 420, with 40 KHz frequency and maximum power of 700 W, the internal dimension (id): 500 × 300 × 150 mm) was utilized for this purpose. The ultrasound extraction was carried out under the following experimental condition: temperature, (50 °C), time, (30 min), solid to solvent ratio (1:8 g/mL) and sonication power (40 KHz and power of 700 W). Hexane was used as the solvent and extraction was performed in duplicate. The extraction condition was obtained from a preliminary extraction [30,31].

### 3.5. Analytical Tests

#### 3.5.1. Fatty Acid Composition (FAC)

Fatty acid methyl esters (FAME) were prepared according to the AOCS Official Method, Ce 2-66 [29] by using 2 M methanolic KOH and hexane. The analysis of fatty acid composition (FAC) was carried out by using an Agilent gas chromatograph (GC) 6890N (Palo Alto, CA, USA) equipped with a flame ionization detector (FID) and a DB-23 capillary column (60 m × 0.25 mm × 0.15 µm) (J&W Scientific, Folsom, CA, USA). A liner (i.d., 0.75 mm, Supelco, Bellefonte, PA, USA) was fixed inside the GC injector to minimize peak widening [32]. The analysis operated under the following conditions: injection volume 0.5 µL, inlet temperature 250 °C, split ratio (1:20). Helium was used as a carrier gas with a flow rate of 0.7 mL/min. Oven temperature was set at 50 °C and held for 1 min at this temperature. Then it was raised to 175 °C with a flow rate of 25 °C/min. In the last step, the temperature reached to 230 °C with the speed of 4 °C/min and held for 5 min at 230 °C. Detector temperature was adjusted at 280 °C. Hydrogen gas and air were used as the detector gases with the flow rate of 40 and 450 mL/min, respectively [33]. The experiment was carried out in duplicate for each sample.

#### 3.5.2. Triacylglycerol (TAG) Profile

##### 3.5.2.1. Qualitative Analysis of Triacylglycerol Composition of Papaya Seed Oil

Qualitative analysis of triacylglycerol profile was carried out by matching the molecular weight of triacylglycerol with a license controller qualification (LCQ) fleet ion trap from a liquid chromatography-mass spectrometer (LC-MS) (Thermo Fisher Scientific, San Jose, CA, USA). LC-MS system was equipped with an auto sampler, C18 column (25 cm × 4.6 mm × 5µm) (Supelco) and mobile phase degasser. For qualitative analysis, 25 µL of the diluted sample was injected. Mobile phase was consisted of three solvents (2-propanol, hexane and acetonitrile). First mobile phase (A) was a mixture of 2-propanol and hexane (5:4); while acetonitrile was the second mobile phase (B) for the gradient condition. Flow rate of mobile phase and column temperature were set at 300 µL/min and 35 °C, respectively. The gradient program was started with 100% mobile phase (B) in the first 50 min of the run. Then, a mixture of both mobile phases (A:B, 50:50) was used for 1 min and only mobile phase A (100%) was pumped for the rest of run [34,35]. A 3D quadrupole ion trap mass analyzer equipped with the atmospheric pressure chemical ionization (APCI) source, and Xcalibur data system was used to perform the data analysis. APCI provides the molecular mass information for semi volatile, medium polar compounds. It is typically used to analyze small molecules up to 2000 Da. APCI can be used in either positive or negative ion polarity mode. For most of compounds, the positive ion mode produces a stronger ion fragmentation [36]. Therefore, APCI source was set at positive ion mode in the present study. Nebulizer pressure, sheath gas pressure and ion sweep gas pressure were set at 3.4, 2.1 and 0.07 bar, respectively. In this experiment, the vaporizing process was carried out at 500 °C for 70 min. The analysis was done in duplicate for each sample.

##### 3.5.2.2. Quantitative Analysis of Triacylglycerol Composition of Papaya Seed Oil

Quantitative analysis of triacylglycerol (TAG) was carried out according to AOCS method (Ce 5b-89) [29] with minor modification to achieve proper peak separation. For triacylglycerol analysis, a high performance liquid chromatography (HPLC) (Waters 600, Waters, Milford, MA, USA) was employed. HPLC system was equipped with a Waters 600 pump, a differential refractometer (RI) detector (Waters 410) and a reverse phase C18 symmetry column (15 cm × 3.9 mm × 5µm). In this experiment, 20 µL of diluted sample 5% (w/v) in acetone was manually injected into the instrument injector. A mixture of acetonitrile and acetone (20:80) was used as the mobile phase, and total run time was set for 50 min [5]. This experiment was performed in duplicate

### 3.6. Statistical Design and Data Analysis

A completely randomized design (CRD) was considered to prepare different experimental treatments. Three different extraction methods were examined: Soxhlet (SXE), solvent extraction (SE) and ultrasound-assisted extraction (UAE). In this study, the effects of solvent extraction conditions (*i.e*., temperature 25 and 50 °C; time 3, 6, 9 and 12 h) on physicochemical properties of papaya seed oil were investigated. The efficiency and suitability of different extraction methods were assessed by determining the recovery yield, fatty acid composition (FAC), and triacylglycerol (TAG) profile of differently extracted papaya seed oils. MINITAB software (version 14) (Minitab Inc., State College, PA, USA) was used to create the experimental design and analyze the data through one way analysis of variance (ANOVA) [37].

## 4. Conclusions

The present study investigated the effect of different extraction methods and conditions on the recovery yield, fatty acid composition and triacylglycerol profile of papaya seed oil. The current research also examined the efficiency of ultrasound-assisted extraction (UAE) as compared to the conventional extraction methods (*i.e*., Soxhlet extraction (SXE), and solvent extraction (SE)) for the recovery of the oil from papaya seeds. This goal was achieved by comparing the extraction yield, FAC and TAG profile of differently extracted papaya seed oils. It was shown that oleic acid (18:0, >70%) and triolein (OOO, >40%) were the predominant fatty acid and triacylglycerol in papaya seed oil. This confirms that papaya seed is a potential source of high oleic oil. However, the current work suggests a further study on the toxicity and safety issue of the crude papaya seed oil. The present study revealed that the ultrasound-assisted extraction was an appropriate technique for recovering the oil from papaya seed. It provided papaya seed oil with desirable fatty acid profile in relatively short extraction times and moderate conditions as compared to different solvent extraction methods. The current research also recommends optimizing the ultrasound conditions in order to achieve papaya seed oil with the most desirable quality.
